# MTHFR and MTRR Polymorphisms Predict Sex-Dependent Psychotic Symptom Improvements, Not Metabolic Changes

**DOI:** 10.3390/ijms27031348

**Published:** 2026-01-29

**Authors:** Sergej Nadalin, Ivan Majdandžić, Jadranka Vraneković, Vjekoslav Peitl, Maja Vilibić, Ante Silić, Dalibor Karlović

**Affiliations:** 1 Department of Psychiatry, General Hospital “Dr. Josip Benčević”, 35000 Slavonski Brod, Croatia; 2 School of Medicine, Catholic University of Croatia, 10000 Zagreb, Croatia; vjekoslav.peitl@gmail.com (V.P.); maja.vilibic@gmail.com (M.V.); ante.silic@gmail.com (A.S.); dalibor.karlovic@gmail.com (D.K.); 3 Department of Anesthesiology, Reanimatology and Intensive Care Medicine, General Hospital “Dr. Josip Benčević”, 35000 Slavonski Brod, Croatia; ivan.majda@gmail.com; 4 Department of Medical Biology and Genetics, Faculty of Medicine, University of Rijeka, 51000 Rijeka, Croatia; jadranka.vranekovic@medri.uniri.hr; 5 Department of Psychiatry, Sestre Milosrdnice University Hospital Center, 10000 Zagreb, Croatia

**Keywords:** antipsychotic agents, DNA methylation, folic acid, homocysteine, metabolism, methylenetetrahydrofolate reductase (MTHFR), methyltetrahydrofolate–homocysteine methyltransferase reductase (MTRR), pharmacogenetics, polymorphism, genetic, psychotic disorders, sex factors

## Abstract

We investigated whether antipsychotic treatment response was influenced by the C677T and A1298C polymorphisms of methylenetetrahydrofolate reductase (MTHFR), and A66G of methyltetrahydrofolate–homocysteine methyltransferase reductase (MTRR)—genes central to folate and homocysteine metabolism and methylation, pathways often altered in schizophrenia patients. To our knowledge, no study has examined associations of C677T and A1298C with changes in schizophrenia symptom severity after antipsychotic treatment, while studies on metabolic outcomes remain sparse and inconsistent. The MTRR A66G has been assessed only once for metabolic parameters—not symptom severity—and sex-stratified analyses are lacking for all polymorphisms. A total of 186 antipsychotic-naïve first-episode or nonadherent chronic psychosis patients and 242 controls were genotyped using PCR-RFLP. Clinical assessments—including Positive and Negative Syndrome Scale (PANSS) scores, PANSS factor scores, and metabolic parameters (fasting plasma lipids and glucose levels, and body mass index)—were conducted at baseline and after 8 weeks. Genotype and allele frequencies did not differ between patients and controls. Significant associations emerged only for symptom changes, specifically within PANSS factor domains, in a sex-dependent manner. Female MTHFR 1298-A allele carriers (AA and AC) showed greater improvement in PANSS negative factor scores, whereas male MTRR 66-G allele carriers (GG and AG) showed reduced improvement in PANSS cognitive factor scores. Effect sizes were strong to very strong, with relatively modest contributions. MTHFR A1298C and MTRR A66G have sex-dependent impacts on symptomatic improvement—but not metabolic outcomes—after antipsychotic treatment. Accordingly, folate–homocysteine genetic markers and sex-specific factors can guide the development of personalized antipsychotic treatment approaches.

## 1. Introduction

Schizophrenia is a severe psychiatric disorder that affects approximately 1–1.5% of the global population [1]. Its etiology is multifactorial, characterized by genetic, epigenetic, and metabolic abnormalities [2,3]. Antipsychotic medications are the mainstay of schizophrenia treatment, improving symptoms and functioning, but atypical antipsychotic medications are associated with metabolic abnormalities [4].

Schizophrenia patients often exhibit elevated plasma homocysteine levels, reduced folates, and altered DNA methylation, both globally and in candidate genes [3,5]. Notably, these changes are implicated in central nervous system neurotransmitter dysfunction [5,6] and metabolic syndrome [7,8]. Elevated homocysteine levels impair neurotransmitter systems by increasing oxidative stress—inducing neurotoxicity via N-methyl-D-aspartate receptor overactivation, and reducing methionine-dependent methylation [9,10]. Altered DNA methylation disrupts neurotransmitter synthesis and synaptic plasticity by modulating gene expression [2,11]. Increased homocysteine contributes to metabolic syndrome by promoting endothelial dysfunction through oxidative stress, and via inflammation and proinflammatory cytokine release [7,12]. Altered DNA methylation can modulate genes promoting inflammation and involved in lipid and glucose metabolism, thereby influencing metabolic syndrome [13,14].

Antipsychotic medications may influence homocysteine levels and DNA methylation, although the evidence is inconsistent [15,16,17,18]. One study reports that olanzapine or risperidone treatment increases homocysteine levels—with corresponding elevations in metabolic parameters (e.g., body mass index (BMI), glucose, triglycerides, and LDL and HDL cholesterol levels) and improvements in positive, negative, and general psychopathology symptoms on the Positive and Negative Syndrome Scale (PANSS) [15]. However, another study shows that risperidone reduces homocysteine levels, which is linked to higher PANSS negative symptoms [16]. Similarly, studies of DNA methylation among patients treated with antipsychotic medications have demonstrated opposite effects on global methylation: clozapine promotes hypomethylation, whereas haloperidol causes hypermethylation [18]. Moreover, clozapine induces widespread hypermethylation and hypomethylation in peripheral leukocytes, with some changes linked to clinical improvement [17].

Genes encoding the enzymes methylenetetrahydrofolate reductase (MTHFR) and methyltetrahydrofolate–homocysteine methyltransferase reductase (MTRR) play central roles in folate and homocysteine metabolism, influencing DNA methylation [3,19]. MTHFR converts 5,10-methylenetetrahydrofolate to 5-methyltetrahydrofolate, a key methyl donor for homocysteine remethylation to methionine [3], while MTRR is essential for remethylation via a cobalamin- and folate-dependent reaction [19]. The C677T and A1298C polymorphisms in MTHFR, and A66G in MTRR, are extensively studied variants [20,21], and MTHFR C677T and A1298C have been widely investigated in schizophrenia etiology [3]. Meta-analyses reveal that the MTHFR 677-T and 1298-C alleles are associated with increased schizophrenia risk, with stronger evidence for the 677-T allele, especially in Asian populations [22,23]. To date, only one study, in a Syrian population, has examined MTRR A66G and schizophrenia etiology, revealing no individual association but borderline risk in combined MTHFR 677-CC/MTRR 66-GG homozygous individuals [19].

Studies have investigated the potential relevance of MTHFR C677T and A1298C in the clinical expression of schizophrenia among chronic patients receiving antipsychotic treatment, with greater focus on metabolic parameters than symptom severity [24,25,26,27,28,29,30,31,32]. In a US study, MTHFR 677-TT patients show greater executive-function deficits and more pronounced negative PANSS symptoms, compared to MTHFR 677-CC patients, while MTHFR A1298C exhibits no effect [24,26]. In one Chinese study, MTHFR 677-T carriers (TT and CT) exhibit greater episodic memory impairment, compared to MTHFR 677-CC individuals [29]. Another Chinese study reported a sex-dependent effect of the MTHFR 677-T allele—with male carriers, but not female carriers, showing more severe PANSS negative symptoms and impaired memory and attention [31]. In a US population, MTHFR 677-T carriers are more vulnerable to metabolic syndrome, compared with MTHFR 677-CC individuals, while MTHFR A1298C has no notable effect [25,28]. Conversely, in a Malaysian sample, MTHFR 677-T carriers are protected against metabolic syndrome, compared to MTHFR 677-CC individuals [30]. In a European sample, MTHFR 1298-CC individuals show higher risk than MTHFR 1298-AA individuals [27]. Finally, a Chinese study reported that MTHFR 677-T carriers have lower total and LDL cholesterol levels, compared with MTHFR 677-CC individuals, while MTHFR 1298-C carriers (CC and AC) with low folate exhibit a higher BMI, despite no direct effect of A1298C on metabolic parameters [32].

Studies of MTHFR C677T, MTHFR A1298C, and MTRR A66G relative to antipsychotic treatment responses are scarce, and are focused on metabolic parameter changes after various antipsychotic medications [33,34,35,36,37]. A European study of chronic patients, recently initiated on or switched to antipsychotic medications, reports that MTHFR 1298-CC patients show greater increases in weight, waist circumference, and plasma glucose after 12 weeks of treatment, compared with MTHFR 1298-AA patients, while MTHFR C677T exhibits no effects [33]. Another European study of chronic and first-episode patients reported that MTHFR 1298-CC patients show greater weight gain after 14 weeks, compared to MTHFR 1298-A carriers (AA and AC), while MTHFR 677-T allele carriers show greater HDL cholesterol increases than MTHFR 677-CC individuals, mainly among first-episode patients [34]. Another European study demonstrates that MTHFR 677-CC first-episode patients experienced greater increases in BMI and waist circumference after 14 weeks, compared to other MTHFR C677T genotypes, while MTHFR A1298C and MTRR A66G show no effects [35]. In a study of two cohorts (Chinese and Spanish) of antipsychotic-naïve first-episode patients, MTHFR 677-CC individuals exhibit greater BMI increases after 10–12 weeks, compared to MTHFR 677-T carriers, while MTHFR A1298C shows no effect [36]. Finally, a Chinese study of first-episode and relapsed patients demonstrates that after 6 weeks of antipsychotic monotherapy—especially with risperidone—MTHFR 677-C carriers (CC and CT) show greater increases of BMI, LDL cholesterol, and waist circumference, compared to MTHFR 677-TT individuals [37].

Since polymorphisms related to folate and methionine metabolism influence homocysteine levels and DNA methylation [3,19], and antipsychotic medications can affect these pathways [15,16,17,18], research on folate- and methionine-metabolism polymorphisms in antipsychotic treatment response is warranted. Studies examining their association with metabolic parameters after antipsychotic therapy remain sparse and inconsistent [33,34,35,36,37]. To our knowledge, no study has examined associations of MTHFR C677T and A1298C polymorphisms with schizophrenia symptom severity following antipsychotic treatment. Moreover, MTRR A66G has been investigated in a single study in relation to metabolic syndrome parameters, but not symptom severity [35], and sex-stratified analyses are largely lacking for all polymorphisms. Schizophrenia exhibits gender-specific differences in clinical presentation and treatment response [38,39], and our previous pharmacogenetic studies demonstrated gene–sex interactions affecting antipsychotic treatment outcomes [40,41]. Therefore, in the present study we hypothesized that MTHFR C677T, MTHFR A1298C, and MTRR A66G polymorphisms might influence antipsychotic treatment-related changes in PANSS psychopathology, plasma lipid and glucose levels, and BMI values, with analyses conducted separately in males and females. Our sample comprised antipsychotic-naïve first-episode patients and nonadherent chronic psychosis patients, and clinical and metabolic assessments were conducted at baseline and after 8 weeks of treatment with various, mainly atypical, antipsychotic medications.

## 2. Results

Male and female patients significantly differed in current age; age of onset; BMI values; and plasma total cholesterol, HDL cholesterol, and triglyceride levels. Compared to females, males exhibited younger age, earlier onset, lower HDL cholesterol levels, higher total cholesterol, higher triglycerides, and higher BMI values (*p* < 0.01 and *p* < 0.05). Both genders exhibited total cholesterol, LDL cholesterol, HDL cholesterol, triglyceride, and glucose levels within the normal range [42]. BMI values were also within the normal range for both genders (Table 1) [43,44]. Body weight was recorded, but significant changes were not observed; therefore, BMI was analyzed as a more standardized and informative indicator [45].

The genotype and allele frequencies of MTHFR polymorphisms were consistent with those in the general European population [23,46], while the MTRR polymorphism distribution aligned with frequencies more commonly observed in southern European populations [47]. The study had statistical power of 80% for detecting a 1.5-fold difference in the frequencies of the MTHFR 677-T, MTHFR 1298-C, and MTRR 66-G alleles between patients and controls. Genotype distributions for MTHFR and MTRR polymorphisms in patients and controls were consistent with Hardy-Weinberg equilibrium. The genotype and allele frequencies of MTHFR and MTRR polymorphisms did not significantly differ between patients and controls, when considering the total group (*p* > 0.05) (Table 2). Additionally, these genotype and allele frequencies did not significantly differ between males and females, within either the patient or control groups (*p* > 0.05).

Among males, PANSS negative symptom score decreases were significantly greater among individuals negative for the MTHFR 677-C allele (TT) compared to MTHFR 677-C allele carriers (CC and CT) (*p* = 0.048, Cohen’s *d* = 0.41), as well as in those carrying the MTHFR 677-T allele (TT and CT) compared to MTHFR 677-CC individuals (*p* = 0.046, Cohen’s *d* = 0.24). The change in glucose levels significantly differed between males carrying the MTHFR 677-T allele (glucose increased) and MTHFR 677-CC homozygous males (glucose decreased) (*p* = 0.031, Cohen’s *d* = 0.58). Compared to MTHFR 677-CC females, females carrying the MTHFR 677-T allele exhibited significantly greater decreases in both PANSS negative symptom scores (*p* = 0.024, Cohen’s *d* = 0.46) and PANSS negative factor scores (*p* = 0.029, Cohen’s *d* = 0.49) (Table 3).

Females carrying the MTHFR 1298-C allele (CC and AC) exhibited greater decreases in PANSS positive symptom scores compared to MTHFR 1298-AA individuals (*p* = 0.028, Cohen’s *d* = 0.54). Moreover, compared to MTHFR 1298-CC females, females carrying the MTHFR 1298-A allele (AA and AC) exhibited significantly greater decreases in PANSS negative symptom scores (*p* = 0.028, Cohen’s *d* = 0.58), PANSS negative factor scores (*p* = 0.008, Cohen’s *d* = 1.05), and PANSS cognitive factor scores (*p* = 0.022, Cohen’s *d* = 0.85) (Table 4).

Compared to MTRR 66-GG individuals, males carrying the MTRR 66-A allele (AA and AG) exhibited greater decreases in PANSS general psychopathology scores (*p* = 0.032, Cohen’s *d* = 0.08), PANSS total symptom scores (*p* = 0.027, Cohen’s *d* = 0.08), PANSS positive factor scores (*p* = 0.046, Cohen’s *d* = 0.46), and PANSS negative factor scores (*p* = 0.016, Cohen’s *d* = 0.54). Compared to males carrying the MTRR 66-G allele (GG and AG), MTRR 66-AA males showed significantly greater decreases in both PANSS depression factor scores (*p* = 0.043, Cohen’s *d* = 0.57) and PANSS cognitive factor scores (*p* = 0.004, Cohen’s *d* = 0.79). Moreover, the change in glucose levels significantly differed between MTRR 66-GG homozygous females (glucose level decreased) and females carrying the MTRR 66-A allele (glucose increased) (*p* = 0.047, Cohen’s *d* = 0.44) (Table 5).

Multiple regression analysis confirmed that MTHFR 1298-A significantly predicted the PANSS negative factor among females (β = −0.27, *p* = 0.048). On the other hand, MTRR 66-G significantly predicted the PANSS cognitive factor among males (β = 0.31, *p* = 0.012). The negative β coefficient for the MTHFR 1298-A allele indicated a significantly greater reduction in PANSS negative factors scores among females carrying this allele (AA and AC), compared to MTHFR 1298-CC females. Similarly, the positive β coefficient for the MTRR 66-G allele indicated significantly smaller decreases in PANSS cognitive factor scores among males carrying MTRR 66-G (GG and AG) compared to MTRR 66-AA males. The MTHFR 1298-A allele accounted for approximately 7.2% of variance in the PANSS negative factor (R^2^ change = 0.072), while the MTRR 66-G allele explained about 9.6% of variance in the PANSS cognitive factor (R^2^ change = 0.096).

Additional exploratory analyses were performed in antipsychotic-naïve first-episode patients and in nonadherent chronic patients, and across subgroups stratified by antipsychotic metabolic-risk profiles (high, medium, low) [48]. Several polymorphism-specific associations with PANSS factor remained significant after multiple regression analyses. In antipsychotic naïve first-episode females, the MTHFR C677T polymorphism predicted changes in the PANSS excitement factor, whereas in nonadherent chronic males, it was associated with changes in the PANSS depressive factor. Additionally, in this same male subgroup, the MTRR A66G polymorphism predicted changes in the PANSS negative factor. When patients were stratified by antipsychotic metabolic-risk profiles, the MTHFR A1298C polymorphism predicted changes in the PANSS excitement factor in males treated with aripiprazole–haloperidol, whereas the MTRR A66G polymorphism predicted changes in the PANSS positive factor in females treated with clozapine–olanzapine. No polymorphism-related associations with metabolic parameters were detected in any subgroup.

Overall, genotype and allele frequencies of MTHFR and MTRR polymorphisms did not differ between patients and controls. Significant sex-dependent associations emerged only in PANSS psychopathology data, specifically within PANSS factor domains, indicating that MTHFR and MTRR polymorphisms influence symptomatic—but not metabolic—response to antipsychotic treatment.

## 3. Discussion

In this study, we aimed to examine the role of MTHFR and MTRR polymorphisms in antipsychotic treatment response, integrating changes in clinical symptomatology and metabolic parameters in a cohort comprising antipsychotic-naïve first-episode patients and nonadherent chronic psychosis patients, stratified by sex. Similar to in many European populations, the frequencies of the MTHFR C677T and A1298C polymorphisms did not differ between patients and controls in our study [22,23], and the MTRR A66G polymorphism also showed no differences between these groups, consistent with a study in the Syrian population [19]. Our primary findings indicate that MTHFR A1298C and MTRR A66G were significantly associated with changes in PANSS psychopathology following antipsychotic treatment. We observed no significant associations of either MTHFR polymorphism, or the MTRR polymorphism, with metabolic-syndrome-related parameters in male or female patients. Specifically, our findings indicate that, compared with MTHFR 1298-CC homozygous females, females carrying the MTHFR 1298-A allele (AA and AC) exhibited greater improvement in PANSS negative factor scores. Additionally, compared with MTRR 66-AA homozygous males, males carrying the MTRR 66-G allele (GG and AG) exhibited reduced improvement in PANSS cognitive factor scores. Although the contributions of MTHFR and MTRR polymorphisms to PANSS psychopathology were relatively modest, effect sizes were strong to very strong, suggesting that both genetic variants may be clinically relevant.

Our data suggest that, among females, the presence of the MTHFR 1298-A allele exerted a protective effect, supporting greater improvement of the PANSS negative factor following antipsychotic treatment (Table 4). On the other hand, among males, the presence of the MTRR 66-G allele exerted a risk effect, leading to reduced improvement in cognitive impairment following treatment (Table 5). Importantly, both findings represent novel associations, extending the current knowledge regarding how polymorphisms related to folate and methionine metabolism can influence schizophrenia symptom severity. The observed association between symptom severity and the MTHFR A1298C polymorphism contradicts previous findings from a US study of chronic schizophrenia patients under antipsychotic therapy, which show no effect of this polymorphism [24]. Additionally, we observed no associations between the MTHFR C677T polymorphism and changes in PANSS psychopathology. This differs from the findings of US studies of chronic patients under antipsychotic therapy, which indicate that the MTHFR 677-TT genotype is a risk factor for more severe PANSS negative symptoms and cognitive impairment [24,26]. Similar risk effects have been identified in Chinese studies of chronic patients under antipsychotic therapy, showing that the MTHFR 677-T allele is a risk factor for more pronounced PANSS negative symptoms and cognitive impairment, with some sex-dependent variations [29,31].

Our present data revealed no associations between MTHFR or MTRR polymorphisms and metabolic-syndrome-related parameters. This is consistent with the findings of European studies examining changes in metabolic parameters after antipsychotic treatment, which demonstrate no effect of the MTHFR C677T polymorphism [33], and of the MTHFR A1298C and MTRR A66G polymorphisms [35]. Similarly, a study conducted in Chinese and Spanish cohorts shows no effect of the MTHFR A1298C polymorphism on BMI [36]. On the other hand, our negative results contrast with the findings of studies among chronic patients under antipsychotic treatment, which have shown that the MTHFR 677-T allele is a risk factor for metabolic syndrome in a US study [25,28], exerts a protective effect in a Malaysian sample [30], and is associated with a protective effect on lipid profiles in a Chinese study [32]. Additional studies report changes related to MTHFR C677T after antipsychotic treatment, with one European study reporting that the MTHFR 677-T allele has a protective effect associated with increased HDL cholesterol levels [34], another European study finding that the MTHFR 677-CC genotype has a risk effect linked to greater increases of BMI and waist circumference [35], a Chinese study showing that the MTHFR 677-C allele has a risk effect associated with greater increases in LDL cholesterol and adiposity measures [37], and a study of Spanish and Chinese cohorts describing a risk effect of the MTHFR 677-CC genotype linked to greater BMI increases [36]. Negative findings regarding the MTHFR A1298C polymorphism contrast with studies in chronic patients under antipsychotic treatment—for example, a European study shows that MTHFR 1298-CC individuals exhibit a greater risk effect for metabolic syndrome [27], while a Chinese study demonstrates that MTHFR 1298-C carriers with low folate exhibit a higher BMI [32]. Other studies have reported changes after antipsychotic treatment that are related to the MTHFR A1298C polymorphism—including two European studies showing that the MTHFR 1298-CC genotype is a risk factor for greater increases in obesity measures and glucose levels [33], and exerts a risk effect for greater weight gain [34].

The present study revealed intriguing differences: the MTHFR C677T polymorphism was not linked to symptom severity, whereas the MTHFR A1298C polymorphism was, and no MTHFR or MTRR polymorphisms were associated with metabolic parameters. These findings differ from previous studies of chronic patients under antipsychotic therapy. Some previous studies report associations of MTHFR C677T with symptom severity [24,26,29,31]. Additionally, MTHFR C677T and A1298C have been associated with metabolic parameters in studies among chronic patients under antipsychotic therapy [25,27,28,30,32], and in studies examining metabolic changes following antipsychotic therapy [33,34,35,36,37]. Variations in study design likely explain the differences between our findings and those in chronic patients on antipsychotic medications. Notably, we evaluated PANSS psychopathology and metabolic parameters at two time-points, whereas prior studies have assessed them only once and have generally not accounted for patient adherence to antipsychotic medications [24,25,26,27,28,29,30,31,32]. Other factors may also contribute to the observed differences between our analysis and other studies investigating changes in metabolic parameters relative to polymorphisms [33,34,35,36,37]. For instance, the effect of the MTHFR C677T polymorphism may vary in accordance with ethnic differences, reflecting population-specific genetic patterns [46,47]. Notably, the MTHFR 677-C allele is generally less frequent in Chinese populations, and is especially rare among Han Chinese individuals, who comprised the samples in the Chinese studies that show that this allele is associated with increased risk of antipsychotic-induced metabolic changes [36,37]. Moreover, the studies have differed in antipsychotic medications. For example, only one Chinese study performed a treatment-specific analysis that included risperidone [37], while the other studies (including our own) did not, and olanzapine was widely prescribed in several studies, whereas its use was minimal in our patient cohort [33,34,35]. Importantly, olanzapine is strongly associated with weight gain, and ranks among the antipsychotic medications imposing the highest metabolic risk [49,50]. Furthermore, variability in the duration of antipsychotic treatment across studies may have influenced weight gain outcomes, with possible implications for metabolic parameters [49,51]. Except for the 6-week Chinese study [37], our 8-week study had one of the shortest treatment durations, with most previous investigations assessing antipsychotic-induced metabolic effects after 10–14 weeks of treatment [33,34,35,36].

Functional studies indicate lower MTHFR activity among MTHFR 1298-C allele carriers, with MTHFR 1298 CC homozygous individuals exhibiting approximately 60% of the activity exhibited by MTHFR 1298-AA individuals [19,52]. In parallel, studies of the MTRR A66G polymorphism suggest that the MTRR 66-G allele is linked to mildly reduced enzyme activity, although the reported enzyme activities have varied [19,53]. Balanced homocysteine metabolism—supported by normal MTHFR and MTRR activities—facilitates DNA methylation and neurotransmitter synthesis, whereas reduced activity of these enzymes leads to elevated homocysteine levels and neurotransmission disruption [21,54]. Therefore, our findings indicating that female MTHFR 1298-A carriers showed greater improvement in PANSS negative factor scores, and that male MTRR 66-G carriers exhibited less improvement in PANSS cognitive factor scores, suggest that schizophrenia patients with preserved MTHFR activity respond more favorably to antipsychotic treatment, while reduced MTRR activity may be associated with poorer therapeutic outcomes. Since antipsychotic medications modulate homocysteine levels [15,16] and DNA methylation [17,18]—both of which are essential for neurotransmission [2,9,10,11]—MTHFR and MTRR gene polymorphisms together may affect pathways and influence the response to antipsychotic treatment.

Intriguingly, although MTHFR C677T appears to exert the strongest effect on enzyme function—with MTHFR 677-TT individuals exhibiting approximately 30% of the enzyme activity shown by MTHFR-CC individuals [19,55]—and is more consistently associated with elevated homocysteine levels [54,56], we did not find that this polymorphism was significantly associated with changes in schizophrenia symptom severity after antipsychotic treatment. This suggests that the association of polymorphisms related to folate and methionine metabolism with the response to antipsychotic treatment may be more complex, and potentially influenced by other yet unknown factors. Potential contributing factors may include diet (i.e., dietary intake of folate and methionine) [21,57] and gene–gene interactions [19].

The presently observed sex-dependent effects of MTHFR and MTRR confirm our prior findings of sex-based differences in antipsychotic treatment response [40,41]. A Chinese study also reports sex-specific effects of the MTHFR 677-T allele, with male carriers showing more pronounced symptom severity and cognitive deficits [31]. Importantly, there are sex-related differences in homocysteine levels and DNA methylation capacity [58,59,60,61]. Estrogen reduces homocysteine levels in females, as supported by findings of lower levels in premenopausal females compared to males, and of increases after menopause [59,61]. Sex-related differences in DNA methylation also occur across tissues: females show higher levels in leukocytes [58], males at specific kidney loci [60], and sex-based differences in brain patterns have been noted but are less characterized [62].

Our study patient cohort was rather heterogeneous regarding antipsychotic medications. Previous studies show that different antipsychotic medications can influence homocysteine levels and DNA methylation [15,16,17,18]. Homocysteine levels and/or DNA methylation may also be influenced by other psychotropic medications that are commonly prescribed to schizophrenia patients [63,64,65,66,67,68]. For instance, mood stabilizers are associated with alterations in both homocysteine metabolism and DNA methylation [63,64,65,66], whereas antidepressants are linked only to DNA methylation changes [67,68].

To address potential clinical and treatment-related heterogeneity in this study, exploratory analyses were performed in antipsychotic-naïve first-episode patients, in nonadherent chronic patients, and in subgroups stratified by antipsychotic metabolic-risk profiles. Several subgroup-specific associations were observed only in PANSS factor domains. However, these associations did not always follow the pattern observed in the main analysis: some polymorphisms showed associations with different PANSS factors, while others were significant despite being nonsignificant in the main analyses. On the other hand, no polymorphism-related associations with metabolic parameters were detected in any subgroup, consistent with the main analyses. Differences between subgroup-specific and main analyses likely reflect small genotype-by-sex and antipsychotic-based subgroup sizes, yet they do not alter the interpretation of the main findings derived from adequately powered sex-stratified analyses of the full cohort.

Overall, the present findings extend existing research on the role of folate- and methionine-metabolism polymorphisms in antipsychotic treatment response. This study is the first to assess the MTHFR C677T, MTHFR A1298C, and MTRR A66G polymorphisms in relation to antipsychotic-induced changes in schizophrenia symptom severity. It also adds to the sparse and inconsistent evidence on treatment-related metabolic outcomes for MTHFR polymorphisms, as well as to the single study to date examining the MTRR A66G variant in relation to metabolic outcomes. Moreover, sex-stratified analyses have been largely absent from previous work on all three polymorphisms, making the inclusion of sex-specific analyses a further contribution of this study. Although only a small number of studies have examined treatment response in relation to folate- and methionine-metabolism polymorphisms, most have been conducted in European populations, rendering the present European cohort particularly relevant for comparison. Taken together, our findings support the potential value of incorporating genetic markers within the folate–homocysteine metabolic pathway, together with sex-specific factors, into personalized approaches to optimizing antipsychotic treatment outcomes.

Limitations of our study include the relatively small sample size, leaving open the possibility that some effects were not detected. Additionally, non-adherence to antipsychotic medications was assessed using anamnestic information. Moreover, we assessed only three polymorphisms, the distributions of which did not differ between patients and controls. Finally, we did not inquire about participants’ dietary habits or intake of specific nutrients (e.g., folates) that could have influenced homocysteine levels, which may have contributed to the variability in our findings.

## 4. Conclusions

We report that the MTHFR A1298C and MTRR A66G polymorphisms had sex-dependent effects on changes in PANSS psychopathology following antipsychotic treatment, showing relatively modest contributions but strong to very strong effect size. On the other hand, neither MTHFR nor MTRR polymorphisms were associated with metabolic-syndrome-related parameters in either sex, a finding that contrasts with previous reports linking MTHFR polymorphisms to antipsychotic-related metabolic changes. There remains a need for replication in larger studies to further clarify these associations, including responses to individual antipsychotic medications, and to account for potential environmental influences, such as dietary intake and gene–gene interactions.

## 5. Patients and Methods

### 5.1. Study Participants

A total of 186 patients with antipsychotic-naïve first-episode psychosis or nonadherent chronic psychosis, and 242 sex- and age-matched control participants, were recruited for this study. All patients were treated at the Department of Psychiatry, University Hospital Centre Sestre Milosrdnice, Zagreb, Croatia, between 2015 and 2022. The patients were consecutively recruited, and were part of a group that had been included in our previous pharmacogenetic analyses [40,41,69,70]. Antipsychotic-naïve first-episode patients and nonadherent chronic patients showed broadly comparable baseline characteristics. PANSS profiles were similar, with nonadherent chronic patients exhibiting slightly higher PANSS positive symptoms, PANSS excitement factor, and consequently a modestly higher total PANSS score. Several metabolic parameters differed statistically, but all values remained within normal reference ranges, indicating no clinically meaningful differences. Given these overall similarities, the two groups were combined in the pharmacogenetic analyses (Supplementary Table S1). Control participants were blood donors, representative of the healthy general population, were typically free from chronic illnesses or regular medication use, and were presumed not to have psychiatric disorders, although this was not formally assessed. Table 1 presents the patients’ baseline characteristics, according to gender.

The inclusion criteria for patients were as follows: (1) Croatian citizenship; (2) age between 18 and 60 years; (3) a confirmed diagnosis using a structured clinical interview according to the according to the Diagnostic and Statistical Manual of Mental Disorders (DSM-V) criteria [71]; (4) for antipsychotic-naïve first-episode patients, no previous treatment with antipsychotic medication; and (5) for nonadherent chronic psychosis patients, prior antipsychotic treatment but currently non-compliant or off depot injections for at least one month, based on auto- and hetero-anamnestic information. The exclusion criteria were as follows: (1) general brain disorder, that might affect cognitive functions (e.g., head injury, Parkinson’s disease, Alzheimer’s disease); (2) current pregnancy or lactation, or history of pregnancy in the past 12 months; (3) comorbid psychoactive substance and/or alcohol use disorder (with exception of nicotine dependence). Among the patients, 163 (87.7%) were diagnosed with schizophrenia, 9 (4.8%) with schizoaffective disorder, and 14 (7.5%) with psychotic disorder not otherwise specified. Both patient groups received 8 weeks of treatment with at least one of the following medications: clozapine (*n* = 84), risperidone (*n* = 45), aripiprazole (*n* = 45), paliperidone (*n* = 40), aripiprazole depot (*n* = 28), haloperidol (*n* = 28), fluphenazine (*n* = 14), and olanzapine (*n* = 13). In the subgroup of nonadherent chronic patients, antipsychotic therapy was generally continued as previously prescribed, with adjustments made as clinically indicated. When anxiolytic or hypnotic medications were not sufficiently effective, many patients were also prescribed antipsychotic promazine (*n* = 64), usually at a lower dose (75–150 mg per day). None of the patients received folic acid or vitamin B supplementation during the study period.

A patient’s mean age at the first hospital admission during which the diagnosis of schizophrenia was used is considered to approximately represent the age at onset. Baseline PANSS psychopathology was recorded within 24 h after hospital admission [72]. PANSS scales were divided into five symptom dimensions (factors): positive (P1, P3, P6, and G9), negative (N2, N3, N4, N6, and G7), excitement (P4, P7, and G1), depression (G2, G3, and G6), and cognitive (G10 and G12) [73,74,75].

This study was approved by the Ethics Committee of Medical Faculty, University of Rijeka, Rijeka (protocol code: 003-08/20-01/15, number: 2170-24-09-8-20-2); the Ethics Committee of Clinical Hospital Center Rijeka, Rijeka (protocol code: 003-05/20-1/82, number: 2170-29-02/1-20-2); and the Ethics Committee of University Hospital Center Sestre Milosrdnice, Zagreb (protocol code: 003-06/20-03/013, number: 251-29-11-20-01-5). All patients received a description of the study’s purpose and methods, and then provided written informed consent. Clinical and laboratory investigations were conducted in accordance with the ethical standards in the latest version of the Declaration of Helsinki.

### 5.2. Genotyping

Genomic DNA was extracted from whole blood samples using the FlexiGene DNA kit 250 (QIAGEN GmbH, Hilden, Germany), according to the manufacturer’s instructions. Genotyping was performed by polymerase chain reaction/restriction fragment length polymorphism analysis, at the Laboratory for Molecular Genetics (Department of Medical Biology and Genetics, Faculty of Medicine, Rijeka). Analyses included the C677T (rs1801133) and A1298C (rs1801131) polymorphisms located in exons 4 and 7 of the MTHFR gene [76], as well as the A66G (rs1801394) polymorphism in exon 2 of the MTRR gene [77], following previously described protocols [78,79,80,81]. Digested PCR fragments were separated by electrophoresis, on a 3% agarose gel containing ethidium bromide, and photographed under UV light with a gel documentation system.

### 5.3. Biochemical Measurements

Venous blood samples were collected in the morning after fasting (12 h), using a standard technique, without venous stasis. Plasma levels of total cholesterol, LDL cholesterol, HDL cholesterol, triglycerides, and glucose were measured using an ARCHITECT c8000 analyzer (Abbott Laboratories, Abbott Park, IL, USA), at the Department of Clinical Chemistry in University Hospital Center Sestre Milosrdnice, Zagreb. Total cholesterol > 5.0 mmol/L, LDL > 3.0 mmol/L, triglycerides > 2.0 mmol/L, and glucose > 6.1 mmol/L were considered elevated, while HDL < 1.0 mmol/L was considered decreased [42]. BMI was calculated as weight (kg) divided by height squared (m^2^). Patients with BMI > 30 were classified as obese, with BMI of 25–30 as overweight but not obese, and with BMI of 18.5–24.9 as exhibiting a normal body weight [43,44].

### 5.4. Statistical Analyses

Statistical analyses were conducted using Statistica for Windows, version 14 (StatSoft, Inc., Tulsa, OK, USA). Nonparametric Mann-Whitney *U* tests or chi-square (χ^2^) tests were used to compare the characteristics of male versus female patients. Mann–Whitney *U* tests were also used to compare baseline characteristics between first-episode and chronic patients. We used χ^2^ tests to compare the observed and expected MTHFR and MTRR genotypes according to Hardy-Weinberg, as well as the MTHFR and MTRR genotype and allele distributions among patients and controls. Genotypes of the C677T and A1298C polymorphisms of MTHFR and the A66G polymorphism of MTRR were analyzed using dominant and recessive models, comparing carriers and non-carriers of each allele as follows: C-positive versus C-negative and T-positive versus T-negative for MTHFR C677T; A-positive versus A-negative and C-positive versus C-negative for MTHFR A1298C; and A-positive versus A-negative and G-positive versus G-negative for MTRR A66G, in accordance with their known functional effects [19,52,53]. Mann-Whitney *U* tests were applied to examine whether MTHFR and MTRR polymorphisms were associated with changes in mean PANSS psychopathology scores, plasma lipid and glucose levels, and BMI values after antipsychotic treatment. Cohen’s *d* method was used to calculate the standardized effect size [82]. Associations that appeared significant (*p* < 0.05) were further examined by multiple regression analyses, controlling for the possible effects of age and number of psychotic episodes. For metabolic parameters (plasma glucose levels), the analyses additionally controlled for antipsychotic type (clozapine and olanzapine) and changes in BMI [83,84]. Significant results (*p* < 0.05) were adjusted using Bonferroni correction. In addition, exploratory analyses were performed using Mann–Whitney *U* tests and multiple regression analyses in antipsychotic-naïve first-episode patients, in nonadherent chronic patients, and across antipsychotic metabolic-risk profiles [48].

## Figures and Tables

**Table 1:** Patients’ characteristics at baseline.

	Males (*N* = 99)	Females (*N* = 87)	*p*
Age	29.0 (24.0–39.0)	40.0 (29.0–55.0)	**<0.001**
Age of onset	23.0 (21.0–29.0)	29.0 (24.0–39.0)	**<0.001**
Antipsychotic-naïve first-episode patients/non-adherent chronic patients	41/58	26/61	0.102
Number of psychotic episodes	2.0 (1.0–4.0)	3.0 (1.0–5.0)	0.131
PANSS positive symptom score	22.0 (18.0–27.0)	23.0 (18.0–26.0)	0.884
PANSS negative symptom score	26.0 (22.0–32.0)	26.0 (21.0–34.0)	0.674
PANSS general psychopathology score	50.0 (44.0–56.0)	48.0 (43.0–56.0)	0.238
PANSS total symptom score	98.0 (87.0–111.0)	97.0 (85.0–111.0)	0.434
PANSS positive factor	13.0 (11.0–16.0)	13.0 (10.0–16.0)	0.445
PANSS negative factor	19.0 (15.0–23.0)	18.0 (15.0–24.0)	0.833
PANSS excitement factor	9.0 (6.0–10.0)	8.0 (6.0–10.0)	0.645
PANSS depression factor	9.0 (7.0–12.0)	9.0 (7.0–11.0)	0.260
PANSS cognitive factor	6.0 (5.0–7.0)	6.0 (5.0–7.0)	0.731
Total cholesterol, mmol/L	4.4 (3.9–5.1)	4.8 (4.1–5.5)	**0.032**
LDL cholesterol, mmol/L	2.7 (2.3–3.2)	2.9 (2.3–3.5)	0.091
HDL cholesterol, mmol/L	1.1 (0.9–0.2)	1.3 (1.1–0.5)	**<0.001**
Triglycerides, mmoL/L	1.2 (0.9–1.8)	1.1 (0.8–1.5)	**0.024**
Glucose, mmol/L	5.2 (4.8–5.8)	5.3 (4.8–5.9)	0.878
Body mass index, kg/m^2^	24.4 (22.8–27.8)	23.2 (20.4–26.6)	**0.011**

Note: Bolded *p*-values indicate statistical significance. Data are presented as median (25th–75th percentile) unless otherwise indicated. Differences were compared using the Mann-Whitney *U* test, with the exception of the ratio of antipsychotic-naïve first-episode patients to nonadherent chronic patients, which was compared by χ^2^ test. PANSS—Positive and Negative Syndrome Scale.

**Table 2:** The frequencies of MTHFR and MTRR genotypes and alleles among patients and controls.

	Genotypes (%)			Alleles (%)	
MTHFR C677T polymorphism (rs1801133)	CC	CT	TT	C	T
Patients (*N* = 186) ^a^	89 (47.9)	73 (39.2)	24 (12.9)	251 (67.5)	121 (32.5)
Controls (*N* = 242) ^b^	110 (45.5)	107 (44.2)	25 (10.3)	327 (67.6)	157 (32.4)
	χ^2^ = 1.35, df = 2, *p* = 0.508			χ^2^ = 0.00, df = 1, *p* = 0.978	
	Genotypes (%)			Alleles (%)	
MTHFR A1298C polymorphism (rs1801131)	AA	AC	CC	A	C
Patients (*N* = 186) ^c^	81 (43.6)	91 (48.9)	14 (7.5)	253 (68.0)	119 (32.0)
Controls (*N* = 242) ^d^	113 (46.7)	105 (43.4)	24 (9.9)	331 (68.4)	153 (31.6)
	χ^2^ = 1.61, df = 2, *p* = 0.447			χ^2^ = 0.01, df = 1, *p* = 0.906	
	Genotypes (%)			Alleles (%)	
MTRR A66G polymorphism (rs1801394)	AA	AG	GG	A	G
Patients (*N* = 186) ^e^	38 (20.4)	95 (51.1)	53 (28.5)	171 (46.0)	201 (54.0)
Controls (*N* = 242) ^f^	48 (19.8)	125 (51.7)	69 (28.5)	221 (45.7)	263 (54.3)
	χ^2^ = 0.03, df = 2, *p* = 0.987			χ^2^ = 0.01, df = 2, *p* = 0.929	

Note: ^a^ *Hardy-Weinberg*: χ^2^ = 2.08, *p* = 0.148; ^b^ *Hardy-Weinberg*: χ^2^ = 0.02, *p* = 0.892; ^c^ *Hardy-Weinberg*: χ^2^ = 2.88, *p* = 0.090; ^d^ *Hardy-Weinberg*: χ^2^ = 0.00, *p* = 0.957; ^e^ *Hardy-Weinberg*: χ^2^ = 0.15, *p* = 0.700; ^f^ *Hardy-Weinberg*: χ^2^ = 0.40, *p* = 0.525.

**Table 3:** PANSS psychopathology data and metabolic syndrome-related parameters after antipsychotic treatment, according to MTHFR C677T polymorphism.

	Males (*N* = 99)	Median (25th–75th)	*p*	Females (*N* = 87)	Median (25th–75th)	*p*
PANSS positive symptom score	CC, CT (*N* = 84) TT (*N* = 15)	−10.0 (−14.0–−4.0) −12.0 (−13.0–−5.0)	0.816	CC, CT (*N* = 78) TT (*N* = 9)	−9.5 (−14.0–−5.0) −11.5 (−15.0–−8.0)	0.482
	TT, CT (*N* = 51) CC (*N* = 48)	−11.0 (−13.0–−6.0) −10.0 (−14.0–−2.0)	0.822	TT, CT (*N* = 42) CC (*N* = 45)	−11.0 (−14.0–−6.0) −9.0 (−14.0–−5.0)	0.585
PANSS negative symptom score	CC, CT (*N* = 84) TT (*N* = 15)	−7.0 (−10.0–−4.0) −13.0 (−15.0–−2.0)	**0.** **048**	CC, CT (*N* = 78) TT (*N* = 9)	−7.5 (−11.0–−4.0) −9.5 (−21.0–−3.0)	0.472
	TT, CT (*N* = 51) CC (*N* = 48)	−9.0 (−11.0–−5.0) −6.0 (−9.0–−4.0)	**0.** **046**	TT, CT (*N* = 42) CC (*N* = 45)	−9.0 (−13.0–−5.0) −7.0 (−10.0–−3.0)	**0.** **024**
PANSS general psychopathology score	CC, CT (*N* = 84) TT (*N* = 15)	−16.0 (−21.0–−11.0) −22.0 (−24.0–−14.0)	0.083	CC, CT (*N* = 78) TT (*N* = 9)	−15.0 (−21.5–−10.0) −18.5 (−26.0–−9.5)	0.380
	TT, CT (*N* = 51) CC (*N* = 48)	−18.0 (−23.0–−11.0) −16.0 (−20.0–−12.0)	0.161	TT, CT (*N* = 42) CC (*N* = 45)	−17.0 (−24.0–−12.0) −12.0 (−20.0–−9.0)	0.068
PANSS total symptom score	CC, CT (*N* = 84) TT (*N* = 15)	−33.0 (−43.0–−24.0) −47.0 (−54.0–−22.0)	0.125	CC, CT (*N* = 78) TT (*N* = 9)	−32.0 (−44.0–−22.5) −39.5 (−57.0–−23.0)	0.300
	TT, CT (*N* = 51) CC (*N* = 48)	−38.0 (−49.0–−26.0) −32.0 (−42.0–−20.5)	0.108	TT, CT (*N* = 42) CC (*N* = 45)	−37.0 (−48.0–−28.0) −28.0 (−42.0–−22.0)	0.056
PANSS positive factor	CC, CT (*N* = 84) TT (*N* = 15)	−7.0 (−9.0–−4.0) −6.0 (−8.0–−5.0)	0.692	CC, CT (*N* = 78) TT (*N* = 9)	−6.0 (−8.0–−4.0) −6.5 (−9.0–−3.5)	0.711
	TT, CT (*N* = 51) CC (*N* = 48)	−7.0 (−9.0–−5.0) −6.0 (−9.0–−4.0)	0.855	TT, CT (*N* = 42) CC (*N* = 45)	−7.0 (−9.0–−4.0) −5.0 (−7.0–−4.0)	0.160
PANSS negative factor	CC, CT (*N* = 84) TT (*N* = 15)	−6.0 (−8.0–−4.0) −10.0 (−11.0–−7.0)	0.061	CC, CT (*N* = 78) TT (*N* = 9)	−6.0 (−9.0–−3.5) −8.5 (−17.0–−2.0)	0.523
	TT, CT (*N* = 51) CC (*N* = 48)	−7.0 (−9.0–−5.0) −5.0 (−9.0–−3.0)	0.084	TT, CT (*N* = 42) CC (*N* = 45)	−8.0 (−11.0–−4.0) −5.0 (−8.0–−2.0)	**0.** **029**
PANSS excitement factor	CC, CT (*N* = 84) TT (*N* = 15)	−4.0 (−5.0–−2.0) −4.0 (−6.0–−3.0)	0.641	CC, CT (*N* = 78) TT (*N* = 9)	−3.0 (−5.0–−2.0) −5.0 (−8.0–−2.0)	0.173
	TT, CT (*N* = 51) CC (*N* = 48)	−4.0 (−6.0–−2.0) −4.0 (−6.0–−2.0)	0.570	TT, CT (*N* = 42) CC (*N* = 45)	−3.0 (−5.0–−2.0) −3.0 (−5.0–−2.0)	0.931
PANSS depression factor	CC, CT (*N* = 84) TT (*N* = 15)	−7.0 (−9.0–−6.0) −8.0 (−10.0–−7.0)	0.219	CC, CT (*N* = 78) TT (*N* = 9)	−7.0 (−9.5–−5.0) −5.5 (−7.0–−4.0)	0.153
	TT, CT (*N* = 51) CC (*N* = 48)	−7.0 (−9.0–−6.0) −7.0 (−10.0–−6.0)	0.600	TT, CT (*N* = 42) CC (*N* = 45)	−7.0 (−9.0–−5.0) −7.0 (−9.0–−5.0)	0.954
PANSS cognitive factor	CC, CT (*N* = 84) TT (*N* = 15)	−1.0 (−3.0–−1.0) −1.0 (−3.0–−1.0)	0.600	CC, CT (*N* = 78) TT (*N* = 9)	−1.0 (−2.0–−1.0) −1.5 (−5.0–−0.5)	0.453
	TT, CT (*N* = 51) CC (*N* = 48)	−1.0 (−3.0–−1.0) −1.0 (−3.0–−1.0)	0.671	TT, CT (*N* = 42) CC (*N* = 45)	−2.0 (−3.0–−1.0) −1.0 (−2.0–−1.0)	0.181
Total cholesterol, mmol/L	CC, CT (*N* = 84) TT (*N* = 15)	0.4 (−0.1–0.9) 0.3 (−0.2–0.7)	0.598	CC, CT (*N* = 78) TT (*N* = 9)	0.4 (−0.4–1.1) 0.1 (−0.2–0.5)	0.781
	TT, CT (*N* = 51) CC (*N* = 48)	0.2 (−0.1–0.7) 0.7 (0.0–1.0)	0.069	TT, CT (*N* = 42) CC (*N* = 45)	0.1 (−0.4–0.8) 0.5 (−0.1–1.1)	0.350
LDL cholesterol, mmol/L	CC, CT (*N* = 84) TT (*N* = 15)	0.2 (−0.1–0.7) 0.2 (−0.1–0.8)	0.983	CC, CT (*N* = 78) TT (*N* = 9)	0.3 (−0.3–0.7) −0.1 (−0.5–0.2)	0.289
	TT, CT (*N* = 51) CC (*N* = 48)	0.2 (−0.1–0.7) 0.3 (−0.1–0.7)	0.891	TT, CT (*N* = 42) CC (*N* = 45)	0.0 (−0.4–0.4) 0.3 (−0.3–0.8)	0.192
HDL cholesterol, mmol/L	CC, CT (*N* = 84) TT (*N* = 15)	0.1 (−0.1–0.2) 0.0 (−0.2–0.2)	0.431	CC, CT (*N* = 78) TT (*N* = 9)	0.0 (−0.1–0.3) 0.0 (−0.2–0.2)	0.619
	TT, CT (*N* = 51) CC (*N* = 48)	0.0 (−0.2–0.2) 0.1 (0.0–0.2)	0.205	TT, CT (*N* = 42) CC (*N* = 45)	0.0 (−0.1–0.2) 0.0 (−0.1–0.3)	0.580
Triglycerides, mmoL/L	CC, CT (*N* = 84) TT (*N* = 15)	0.2 (−0.2–0.6) 0.2 (−0.8–0.7)	0.727	CC, CT (*N* = 78) TT (*N* = 9)	0.1 (−0.2–0.6) 0.4 (0.2–1.1)	0.144
	TT, CT (*N* = 51) CC (*N* = 48)	0.2 (−0.4–0.5) 0.2 (−0.1–0.7)	0.255	TT, CT (*N* = 42) CC (*N* = 45)	0.2 (−0.1–0.5) 0.1 (−0.2–0.7)	0.895
Glucose, mmol/L	CC, CT (*N* = 84) TT (*N* = 15)	0.1 (−0.6–0.8) 0.1 (−0.5–0.7)	0.659	CC, CT (*N* = 78) TT (*N* = 9)	0.0 (−0.6–0.6) 0.5 (−0.6–2.3)	0.244
	TT, CT (*N* = 51) CC (*N* = 48)	0.1 (−0.4–1.0) −0.1 (−0.9–0.5)	**0.** **031**	TT, CT (*N* = 42) CC (*N* = 45)	0.0 (−0.6–0.8) 0.0 (−0.7–0.6)	0.588
Body mass index, kg/m^2^	CC, CT (*N* = 84) TT (*N* = 15)	0.6 (−0.3–1.9) −0.6 (−0.9–1.2)	0.321	CC, CT (*N* = 78) TT (*N* = 9)	0.7 (−0.7–1.5) 1.1 (−0.3–1.8)	0.555
	TT, CT (*N* = 51) CC (*N* = 48)	0.6 (−0.3–2.0) 0.6 (−0.3–1.6)	0.875	TT, CT (*N* = 42) CC (*N* = 45)	0.7 (−0.9–1.4) 1.1 (−0.3–1.7)	0.237

Note: Bolded *p*-values indicate statistical significance. Data are presented as median (25th–75th percentile). Differences were compared using the Mann-Whitney *U* test. PANSS—Positive and Negative Syndrome Scale. Negative values indicate decreases after antipsychotic treatment. Positive values indicate increases after antipsychotic treatment.

**Table 4:** PANSS psychopathology data and metabolic syndrome-related parameters after antipsychotic treatment, according to MTHFR A1298C polymorphism.

	Males (*N* = 99)	Median (25th–75th)	*p*	Females (*N* = 87)	Median (25th–75th)	*p*
PANSS positive symptom score	AA, AC (*N* = 92) CC (*N* = 7)	−10.5 (−14.0–−6.0) −7.0 (−11.0–−2.0)	0.533	AA, AC (*N* = 80) CC (*N* = 7)	−10.0 (−14.0–−6.0) −8.0 (−13.0–−5.0)	0.636
	CC, AC (*N* = 59) AA (*N* = 40)	−10.0 (−14.0–−2.0) −11.0 (−13.0–−6.5)	0.688	CC, AC (*N* = 44) AA (*N* = 43)	−11.0 (−15.5–−7.5) −8.0 (−13.5–−3.5)	**0.** **028**
PANSS negative symptom score	AA, AC (*N* = 92) CC (*N* = 7)	−7.5 (−11.0–−4.0) −7.5 (−10.0–−5.0)	0.903	AA, AC (*N* = 80) CC (*N* = 7)	−8.0 (−12.0–−4.0) −3.0 (−5.0–0.0)	**0.** **036**
	CC, AC (*N* = 59) AA (*N* = 40)	−6.0 (−10.0–−4.0) −9.0 (−11.0–−5.5)	0.078	CC, AC (*N* = 44) AA (*N* = 43)	−7.5 (−11.0–−4.5) −7.5 (−12.0–−3.5)	0.950
PANSS general psychopathology score	AA, AC (*N* = 92) CC (*N* = 7)	−16.5 (−22.0–−11.0) −18.0 (−19.0–−14.0)	0.692	AA, AC (*N* = 80) CC (*N* = 7)	−16.0 (−23.0–−10.0) −12.0 (−12.0–−12.0)	0.120
	CC, AC (*N* = 59) AA (*N* = 40)	−16.5 (−21.0–−11.0) −17.5 (−22.0–−13.0)	0.337	CC, AC (*N* = 44) AA (*N* = 43)	−17.0 (−22.0–−12.0) −14.0 (−22.5–−7.5)	0.180
PANSS total symptom score	AA, AC (*N* = 92) CC (*N* = 7)	−34.5 (−44.0–−23.0) −32.5 (−40.0–−27.0)	0.955	AA, AC (*N* = 80) CC (*N* = 7)	−34.0 (−46.0–−23.0) −23.0 (−31.0–−22.0)	0.084
	CC, AC (*N* = 59) AA (*N* = 40)	−32.5 (−43.5–−22.5) −37.5 (−49.0–−24.0)	0.448	CC, AC (*N* = 44) AA (*N* = 43)	−35.5 (−44.0–−26.5) −29.5 (−44.5–−16.0)	0.210
PANSS positive factor	AA, AC (*N* = 92) CC (*N* = 7)	−7.0 (−9.0–−5.0) −5.0 (−9.0–−2.0)	0.533	AA, AC (*N* = 80) CC (*N* = 7)	−6.0 (−8.0–−4.0) −6.5 (−8.0–−3.0)	0.879
	CC, AC (*N* = 59) AA (*N* = 40)	−7.0 (−9.0–−5.0) −6.0 (−7.5–−4.0)	0.128	CC, AC (*N* = 44) AA (*N* = 43)	−6.0 (−8.0–−5.0) −5.5 (−8.0–−3.0)	0.307
PANSS negative factor	AA, AC (*N* = 92) CC (*N* = 7)	−7.0 (−9.0–−4.0) −7.0 (−8.0–−3.0)	0.916	AA, AC (*N* = 80) CC (*N* = 7)	−6.5 (−10.0–−4.0) −1.0 (−3.0–−1.0)	**0.** **008**
	CC, AC (*N* = 59) AA (*N* = 40)	−6.5 (−9.0–−3.0) −7.0 (−9.0–−5.0)	0.645	CC, AC (*N* = 44) AA (*N* = 43)	−6.0 (−9.0–−3.0) −6.5 (−10.0–−4.0)	0.549
PANSS excitement factor	AA, AC (*N* = 92) CC (*N* = 7)	−4.0 (−6.0–−2.0) −2.5 (−5.0–−1.0)	0.199	AA, AC (*N* = 80) CC (*N* = 7)	−3.5 (−5.0–−2.0) −2.0 (−4.0–−2.0)	0.328
	CC, AC (*N* = 59) AA (*N* = 40)	−4.0 (−5.0–−2.0) −4.0 (−6.0–−2.0)	0.299	CC, AC (*N* = 44) AA (*N* = 43)	−4.0 (−6.0–−2.0) −3.0 (−5.0–−2.0)	0.183
PANSS depression factor	AA, AC (*N* = 92) CC (*N* = 7)	−7.0 (−9.0–−6.0) −8.5 (−12.0–−6.0)	0.422	AA, AC (*N* = 80) CC (*N* = 7)	−7.0 (−9.0–−5.0) −7.5 (−11.0–−6.0)	0.662
	CC, AC (*N* = 59) AA (*N* = 40)	−7.0 (−10.0–−6.0) −7.0 (−9.0–−6.0)	0.875	CC, AC (*N* = 44) AA (*N* = 43)	−8.0 (−10.0–−6.0) −7.0 (−8.0–−4.5)	0.118
PANSS cognitive factor	AA, AC (*N* = 92) CC (*N* = 7)	−1.0 (−3.0–−1.0) −1.5 (−2.0–0.0)	0.325	AA, AC (*N* = 80) CC (*N* = 7)	−1.0 (−2.0–−1.0) 0.0 (−1.0–0.0)	**0.** **022**
	CC, AC (*N* = 59) AA (*N* = 40)	−1.5 (−3.0–−1.0) −1.0 (−3.0–−1.0)	0.645	CC, AC (*N* = 44) AA (*N* = 43)	−1.0 (−2.0–−1.0) −1.0 (−2.0–−1.0)	0.965
Total cholesterol, mmol/L	AA, AC (*N* = 92) CC (*N* = 7)	0.5 (−0.1–0.9) 0.3 (−0.1–0.4)	0.614	AA, AC (*N* = 80) CC (*N* = 7)	0.3 (−0.3–0.9) −0.3 (−0.5–0.8)	0.332
	CC, AC (*N* = 59) AA (*N* = 40)	0.4 (−0.1–0.9) 0.5 (−0.1–0.9)	0.987	CC, AC (*N* = 44) AA (*N* = 43)	0.4 (−0.3–1.1) 0.2 (−0.4–0.7)	0.443
LDL cholesterol, mmol/L	AA, AC (*N* = 92) CC (*N* = 7)	0.2 (−0.1–0.7) 0.2 (−0.1–0.7)	0.933	AA, AC (*N* = 80) CC (*N* = 7)	0.2 (−0.3–0.7) −0.3 (−0.4–0.3)	0.342
	CC, AC (*N* = 59) AA (*N* = 40)	0.2 (−0.1–0.7) 0.3 (−0.1–0.8)	0.761	CC, AC (*N* = 44) AA (*N* = 43)	0.3 (−0.3–0.7) 0.0 (−0.4–0.6)	0.322
HDL cholesterol, mmol/L	AA, AC (*N* = 92) CC (*N* = 7)	0.1 (−0.1–0.2) 0.1 (−0.1–0.1)	0.707	AA, AC (*N* = 80) CC (*N* = 7)	0.0 (−0.1–0.3) 0.0 (−0.1–0.2)	0.977
	CC, AC (*N* = 59) AA (*N* = 40)	0.1 (−0.1–0.2) 0.1 (−0.2–0.3)	0.916	CC, AC (*N* = 44) AA (*N* = 43)	0.0 (−0.1–0.3) 0.0 (−0.1–0.3)	0.707
Triglycerides, mmoL/L	AA, AC (*N* = 92) CC (*N* = 7)	0.2 (−0.2–0.7) 0.0 (−0.8–0.6)	0.491	AA, AC (*N* = 80) CC (*N* = 7)	0.2 (−0.2–0.7) −0.1 (−0.1–0.2)	0.384
	CC, AC (*N* = 59) AA (*N* = 40)	0.2 (−0.2–0.6) 0.2 (−0.6–0.7)	0.727	CC, AC (*N* = 44) AA (*N* = 43)	0.1 (−0.1–0.7) 0.2 (−0.2–0.7)	0.970
Glucose, mmol/L	AA, AC (*N* = 92) CC (*N* = 7)	0.1 (−0.6–0.7) 0.2 (−0.1–1.3)	0.414	AA, AC (*N* = 80) CC (*N* = 7)	0.0 (−0.6–0.6) 0.5 (−0.8–1.6)	0.556
	CC, AC (*N* = 59) AA (N = 40)	0.3 (−0.4–0.9) 0.1 (−0.6–0.7)	0.437	CC, AC (*N* = 44) AA (*N* = 43)	−0.1 (−0.6–0.3) 0.2 (−0.6–0.8)	0.110
Body mass index, kg/m^2^	AA, AC (*N* = 92) CC (*N* = 7)	0.6 (−0.3 –1.9) 1.3 (0.3 –2.1)	0.491	AA, AC (*N* = 80) CC (*N* = 7)	0.9 (−0.7–1.5) −0.8 (−1.2–1.7)	0.601
	CC, AC (*N* = 59) AA (*N* = 40)	0.5 (−0.3 –1.7) 1.0 (−0.6 –2.0)	0.746	CC, AC (*N* = 44) AA (*N* = 43)	1.0 (−0.4–1.7) 0.7 (−0.7–1.5)	0.731

Note: Bolded *p*-values indicate statistical significance. Data are presented as median (25th–75th percentile). Differences were compared using the Mann-Whitney *U* test. PANSS—Positive and Negative Syndrome Scale. Negative values indicate decreases after antipsychotic treatment. Positive values indicate increases after antipsychotic treatment.

**Table 5:** PANSS psychopathology data and metabolic syndrome-related parameters after antipsychotic treatment, according to MTRR A66G polymorphism.

	Males (*N* = 99)	Median (25th–75th)	*p*	Females (*N* = 87)	Median (25th–75th)	*p*
PANSS positive symptom score	AA, AG (*N* = 66) GG (*N* = 33)	−11.0 (−14.0–−7.0) −9.0 (−12.0–−4.0)	0.196	AA, AG (*N* = 65) GG (*N* = 22)	−9.0 (−13.0–−4.5) −14.0 (−15.0–−7.0)	0.161
	GG, AG (*N* = 80) AA (*N* = 19)	−10.0 (−13.0–−4.0) −12.0 (−14.0–−5.0)	0.851	GG, AG (*N* = 67) AA (*N* = 20)	−10.0 (−15.0–−7.0) −8.0 (−12.0–−3.0)	0.169
PANSS negative symptom score	AA, AG (*N* = 66) GG (*N* = 33)	−8.0 (−12.0–−5.0) −6.0 (−9.0–−3.0)	0.067	AA, AG (*N* = 65) GG (*N* = 22)	−7.0 (−11.0–−3.5) −8.5 (−12.5–4.5)	0.386
	GG, AG (*N* = 80) AA (*N* = 19)	−7.0 (−10.0–−4.0) −11.0 (−15.0–−4.0)	0.285	GG, AG (*N* = 67) AA (*N* = 20)	−8.0 (−12.0–−5.0) −4.5 (−11.0–−1.0)	0.299
PANSS general psychopathology score	AA, AG (*N* = 66) GG (*N* = 33)	−18.0 (−23.0–−12.0) −14.0 (−18.0–−11.0)	**0.** **032**	AA, AG (*N* = 65) GG (*N* = 22)	−15.5 (−21.5–−9.5) −17.0 (−23.0–−11.0)	0.623
	GG, AG (*N* = 80) AA (*N* = 19)	−16.0 (−21.0–−12.0) −21.0 (−24.0–−8.0)	0.315	GG, AG (*N* = 67) AA (*N* = 20)	−15.0 (−22.0–−11.0) −13.0 (−20.0–−6.0)	0.356
PANSS total symptom score	AA, AG (*N* = 66) GG (*N* = 33)	−37.0 (−49.0–−24.0) −29.0 (−39.0–−21.0)	**0.** **027**	AA, AG (*N* = 65) GG (*N* = 22)	−31.5 (−43.0–−24.0) −39.5 (−48.0–−21.5)	0.291
	GG, AG (*N* = 80) AA (*N* = 19)	−33.0 (−43.0–−23.0) −44.0 (−49.0–−24.0)	0.224	GG, AG (*N* = 67) AA (*N* = 20)	−33.0 (−44.0–−26.0) −25.5 (−39.0–−13.0)	0.273
PANSS positive factor	AA, AG (*N* = 66) GG (*N* = 33)	−7.0 (−9.0–−5.0) −5.0 (−8.0–−4.0)	**0.** **046**	AA, AG (*N* = 65) GG (*N* = 22)	−5.5 (−7.5–−4.0) −7.0 (−9.5–−4.0)	0.144
	GG, AG (*N* = 80) AA (*N* = 19)	−6.0 (−9.0–−4.0) −8.0 (−9.0–−6.0)	0.251	GG, AG (*N* = 67) AA (*N* = 20)	−6.0 (−8.0–−4.0) −4.5 (−8.0–−2.0)	0.155
PANSS negative factor	AA, AG (*N* = 66) GG (*N* = 33)	−7.0 (−10.0–−5.0) −6.0 (−7.0–−2.0)	**0.** **016**	AA, AG (*N* = 65) GG (*N* = 22)	−6.0 (−9.0–−3.5) −7.0 (−10.0–−3.0)	0.615
	GG, AG (*N* = 80) AA (*N* = 19)	−6.0 (−8.0–−3.0) −9.0 (−11.0–−5.0)	0.057	GG, AG (*N* = 67) AA (*N* = 20)	−6.0 (−10.0–−4.0) −3.5 (−9.0–−1.0)	0.133
PANSS excitement factor	AA, AG (*N* = 66) GG (*N* = 33)	−4.0 (−6.0–−3.0) −4.0 (−5.0–−1.0)	0.174	AA, AG (*N* = 65) GG (*N* = 22)	−3.0 (−5.0–−2.0) −5.0 (−5.5–−2.0)	0.301
	GG, AG (*N* = 80) AA (*N* = 19)	−4.0 (−5.0–−2.0) −4.0 (−6.0–−4.0)	0.308	GG, AG (*N* = 67) AA (*N* = 20)	−4.0 (−5.0–−2.0) −2.0 (−3.0–−2.0)	0.054
PANSS depression factor	AA, AG (*N* = 66) GG (*N* = 33)	−7.0 (−10.0–−6.0) −7.0 (−9.0–−6.0)	0.554	AA, AG (*N* = 65) GG (*N* = 22)	−7.0 (−10.0–−5.0) −7.0 (−8.5–−4.0)	0.540
	GG, AG (*N* = 80) AA (*N* = 19)	−7.0 (−9.0–−6.0) −9.0 (−11.0–−7.0)	**0.** **043**	GG, AG (*N* = 67) AA (*N* = 20)	−7.0 (−10.0–−5.0) −7.0 (−9.0–−5.0)	0.940
PANSS cognitive factor	AA, AG (*N* = 66) GG (*N* = 33)	−2.0 (−3.0–−1.0) −1.0 (−2.0–1.0)	0.085	AA, AG (*N* = 65) GG (*N* = 22)	−1.0 (−2.0–−1.0) −2.0 (−3.0–1.0)	0.217
	GG, AG (*N* = 80) AA (*N* = 19)	−1.0 (−2.0–−1.0) −3.0 (−3.0–−2.0)	**0.** **004**	GG, AG (*N* = 67) AA (*N* = 20)	−1.0 (−2.0–−1.0) −1.0 (−2.0–0.0)	0.419
Total cholesterol, mmol/L	AA, AG (*N* = 66) GG (*N* = 33)	0.5 (−0.1–0.9) 0.1 (−0.1–0.8)	0.272	AA, AG (*N* = 65) GG (*N* = 22)	0.1 (−0.4–0.9) 0.5 (−0.1–1.3)	0.267
	GG, AG (*N* = 80) AA (*N* = 19)	0.4 (−0.1–0.9) 0.3 (−0.2–1.0)	0.832	GG, AG (*N* = 67) AA (*N* = 20)	0.2 (−0.3–0.8) 0.6 (−0.4–1.1)	0.609
LDL cholesterol, mmol/L	AA, AG (*N* = 66) GG (*N* = 33)	0.3 (−0.1–0.9) 0.1 (−0.1–0.4)	0.482	AA, AG (*N* = 65) GG (*N* = 22)	0.2 (−0.3–0.7) 0.1 (−0.4–0.8)	0.694
	GG, AG (*N* = 80) AA (*N* = 19)	0.2 (−0.1–0.7) 0.4 (−0.3–0.9)	0.766	GG, AG (*N* = 67) AA (*N* = 20)	0.0 (−0.3–0.6) 0.4 (−0.2–0.8)	0.327
HDL cholesterol, mmol/L	AA, AG (*N* = 66) GG (*N* = 33)	0.1 (−0.1–0.2) 0.1 (−0.1–0.2)	0.707	AA, AG (*N* = 65) GG (*N* = 22)	0.0 (−0.1–0.3) 0.2 (0.0–0.3)	0.115
	GG, AG (*N* = 80) AA (*N* = 19)	0.1 (−0.1–0.2) −0.1 (−0.2–0.2)	0.087	GG, AG (*N* = 67) AA (*N* = 20)	0.0 (−0.1–0.2) 0.0 (−0.1–0.4)	0.820
Triglycerides, mmoL/L	AA, AG (*N* = 66) GG (*N* = 33)	0.2 (−0.2–0.7) 0.1 (−0.4–0.5)	0.305	AA, AG (*N* = 65) GG (*N* = 22)	0.2 (−0.2–0.6) 0.1 (−0.2–0.7)	0.758
	GG, AG (*N* = 80) AA (*N* = 19)	0.2 (−0.2–0.5) 0.4 (−0.3–0.9)	0.361	GG, AG (*N* = 67) AA (*N* = 20)	0.3 (−0.1–0.7) 0.0 (−0.2–0.2)	0.079
Glucose, mmol/L	AA, AG (*N* = 66) GG (*N* = 33)	0.1 (−0.5–1.0) −0.1 (−0.7–0.3)	0.119	AA, AG (*N* = 65) GG (*N* = 22)	0.2 (−0.6–0.7) −0.6 (−0.7–0.2)	**0.0** **47**
	GG, AG (*N* = 80) AA (*N* = 19)	0.1 (−0.5–0.9) −0.4 (−0.6–0.6)	0.427	GG, AG (*N* = 67) AA (*N* = 20)	0.0 (−0.6–0.7) 0.2 (−0.6–0.7)	0.550
Body mass index, kg/m^2^	AA, AG (*N* = 66) GG (*N* = 33)	0.9 (−0.3 –2.0) 0.4 (−1.6 –1.7)	0.063	AA, AG (*N* = 65) GG (*N* = 22)	1.0 (−0.7–1.7) 0.3 (−0.7–1.2)	0.351
	GG, AG (*N* = 80) AA (*N* = 19)	0.6 (−0.3 –2.0) 1.1 (−0.9 –1.3)	0.650	GG, AG (*N* = 67) AA (*N* = 20)	1.0 (−0.7–1.7) 0.7 (−0.7–1.4)	0.667

Note: Bolded *p*-values indicate statistical significance. Data are presented as median (25th–75th percentile). Differences were compared using the Mann-Whitney *U* test. PANSS—Positive and Negative Syndrome Scale. Negative values indicate decreases after antipsychotic treatment. Positive values indicate increases after antipsychotic treatment.

## Data Availability

The data presented in this study are available on request from the corresponding author. The data are not publicly available due to GDPR regulations. Data is unavailable due to privacy or ethical restrictions.

## Supplementary Materials

The following supporting information can be downloaded at: https://www.mdpi.com/article/10.3390/ijms27031348/s1.
