# Supplementary material for: MTHFR and MTRR Polymorphisms Predict Sex-Dependent Psychotic Symptom Improvements, Not Metabolic Changes
Source: Int J Mol Sci. 2026 Jan 29;27(3):1348. doi: 10.3390/ijms27031348 (PMC12898146; doi:10.3390/ijms27031348)
Supplement: Supplementary file 1 [file ijms-27-01348-s001.zip › ijms-4080811-supplementary.pdf]

Supplementary Table S1.
Baseline characteristics of antipsychotic-naïve first-episode patients and nonadherent chronic patients.

	Antipsychotic-naïve first-episode patients (N = 67)	Nonadherent chronic patients (N = 119)	*p*
PANSS positive symptom score	21.0 (17.0 – 23.0)	23.0 (19.0 – 28.0)	**<0.01**
PANSS negative symptom score	25.0 (20.0 – 29.5)	27.0 (22.0 – 34.0)	0.059
PANSS general psychopathology score	48.0 (43.0 – 55.5)	50.0 (44.0 – 56.0)	0.221
PANSS total symptom score	94.0 (81.0 – 106.0)	100.0 (87.0 – 114.0)	**0.023**
PANSS positive factor	13.0 (11.0 – 15.0)	13.0 (10.0 – 16.0)	0.276
PANSS negative factor	19.0 (15.0 – 22.0)	18.0 (16.0 – 24.0)	0.271
PANSS excitement factor	8.0 (6.0 – 9.0)	9.0 (7.0 – 10.0)	**0.016**
PANSS depression factor	9.0 (7.0 – 12.0)	8.5 (7.0 – 11.0)	0.317
PANSS cognitive factor	6.0 (5.0 – 6.0)	6.0 (5.0 – 7.0)	0.541
Total cholesterol, mmol/L	4.2 (3.8 – 4.7)	4.8 (4.3 – 5.6)	**<0.001**
LDL cholesterol, mmol/L	2.4 (2.1 – 2.9)	2.9 (2.5 – 3.5)	**<0.001**
HDL cholesterol, mmol/L	1.2 (1.0 – 1.4)	1.1 (1.0 –1.4)	0.867
Triglycerides, mmoL/L	1.1 (0.8 – 1.3)	1.2 (0.9 – 1.6)	0.064
Glucose, mmol/L	5.1 (4.6 – 5.7)	5.4 (5.0 – 6.0)	**<0.001**
Body mass index, kg/m^2	23.0 (20.4 – 25.5)	24.3 (22.6 – 27.7)	**<0.01**

Note: Bolded *p*-values indicate statistical significance. Data are presented as median (25th–75th percentile). Differences were compared using the Mann-Whitney *U* test. PANSS—Positive and Negative Syndrome Scale.
